# Are you listening?

**DOI:** 10.1172/JCI196864

**Published:** 2025-07-15

**Authors:** Amanda N. Pinski

**Affiliations:** Washington University in St. Louis, School of Medicine, St. Louis, Missouri, USA.

Podcasts are everywhere — are you listening? There’s a podcast for just about anything from solo traveling and food history to language learning and sustainable gardening. Biomedical researchers consume a substantial part of this virtual space, publishing a plethora of podcasts ranging from microbiology to clinical psychology. Some podcasts are even purely dedicated to discussing the daily trials and tribulations in the lab. Everyone is talking about the latest research in *Nature* and the epically failed experiment they performed yesterday. But what does this really mean for us as researchers? Do we really need this extra chatter in our already verbally cluttered world? The short answer is yes. Let me tell you why.

My career as a virologist began with podcasts. Prior to listening to *This Week in Virology*, I was an undergraduate unsure of what I wanted to study during my PhD training. This was, frankly, because I didn’t know much about research beyond what I had read in textbooks. All I knew was that I wanted to do experiments and change the world. I didn’t know how to do that or what questions to ask, let alone who sat at the table in each field. However, podcasts allowed me to sit at various tables with full anonymity and unlimited curiosity. No matter where I was and what I was doing, I could listen to scientists in diverse fields talk about their research and careers. All I needed was internet access. This gave me a full picture of what it was like to be a research scientist. Specifically, I learned how past research inspired current investigations; I virtually “met” and heard the career stories of scientists from across the world; and I gained an invaluable understanding of all the hats researchers wear inside and outside the lab. I also learned to not just absorb information, but also critically evaluate it, which is something I was never taught in the classroom. This was the immersive learning experience I needed to determine if research was right for me, an experience undoubtedly shared with many other young scientists. Thus, after a few intriguing episodes about the 2013 West Africa Ebola virus epidemic, virology claimed my interest, and I started the journey I’m on today.

Yet podcasts are so much more than highly accessible, geographically unrestricted outlets for aspiring scientists. They’re a multifaceted tool for scientists of all stages, backgrounds, and resources. First, constant access to diverse thoughts on current topics may very well help you troubleshoot that assay or write that grant you’ve been working on for months, because you can think outside the box figuratively and literally. And that’s just because you decided to listen to something on your morning commute. Subsequently, your comfort with diving into different fields can foster much-needed interdisciplinary interactions to answer bigger questions that have been unanswered for decades, like chronic disease etiology. In the end, regardless of whether the interactions are in-person or not, this results in you having a more highly connected research community that may span the globe. Engaging with research via dialogue becomes increasingly important as we navigate an era inundated with scientific (mis)information: Only direct discussion with the original sources and their accompanying experts can truly help us navigate this crisis. We might not agree with the perspectives we hear on podcasts about this, but they offer us opportunities to practice communicating more than just what we printed in our *Cell* article. We ultimately walk away from each listening session with better critical thinking, networking, and communication skills.

But most of all, podcasts are a way to connect and support researchers on a personal, not just educational and professional, level. Being a researcher is tough. We don’t realize until we’re knee-deep that failure and struggle are routine, predictability is rare, and external factors can work against us. We can easily feel isolated, since emphasis is put on our scientific contributions rather than our well-being. No one seems to talk about it much either. But hearing podcast hosts discuss their stress during a period of continually failed experiments and guest speakers describe how continual manuscript rejection triggered their depression opens the conversation to how we can improve our research by starting with both the individual *and* how and what we communicate with each other. After all, we can only do good research when we’re in the right head space to think through challenges and celebrate successes together. Having a faceless environment to start this conversation is vital for us to sustain a strong, inclusive research community.

Therefore, podcasts serve as tools to teach, inspire, connect, and help us grow as an integrated community that will continue to thrive as creative thinkers, excellent communicators, and supportive colleagues. So, is all this chatter worth it? Absolutely. I hope you continue listening.

## Version 1 07/15/2025

Electronic publication

